# Examination of cardiac functions during acute attack and remission period in children with familial Mediterranean fever

**DOI:** 10.1007/s00431-024-05570-y

**Published:** 2024-04-26

**Authors:** Yusuf Gunay, Fatih Karagozlu, Sanem Gemici, Seyma Sukran Yilmaz, Sezgin Sahin, Kenan Barut, Ozgur Kasapcopur, Reyhan Dedeoglu

**Affiliations:** 1 Department of Pediatrics, Cerrahpasa Faculty of Medicine, Istanbul University-Cerrahpasa, Istanbul, Turkey; 2 Department of Pediatric Cardiology, Cerrahpasa Faculty of Medicine, Istanbul University-Cerrahpasa, Istanbul, Turkey; 3 Department of Pediatric Rheumatology, Cerrahpasa Faculty of Medicine, Istanbul University-Cerrahpasa, Istanbul, Turkey

**Keywords:** Familial Mediterranean fever attack, Speckle tracking echocardiography, Cardiac functions

## Abstract

Familial Mediterranean fever (FMF) is an autosomal recessive autoinflammatory disease characterized by recurring serosal inflammation. Cardiac involvement in FMF commonly manifests as pericarditis and pericardial effusion; however, there is limited research on myocardial function. This study aimed to assess cardiac functions during active inflammation and remission periods of FMF patients and investigate the cardiac effects of inflammation during the attack period. Thirty-eight FMF patients without additional cardiac diseases were included in the study. Demographic characteristics, clinical symptoms, family history, and *MEFV* gene analysis results were obtained retrospectively. Blood tests, blood pressure measurements, electrocardiogram evaluations, conventional echocardiography, and speckle tracking echocardiography were performed during the attack and remission periods. Disease severity was assessed using the Pras scoring system. During the attack period, FMF patients exhibited significantly higher leukocyte count, neutrophil count, C-reactive protein, and erythrocyte sedimentation rate compared to the remission period (*p* < 0.005). Speckle tracking echocardiography revealed decreased function in the inferior segments of the left ventricle during the attack period (*p* < 0.005). Right ventricular function was more affected in the moderate disease group. FMF patients with lymphopenia during the attack demonstrated more impaired right ventricular function compared to those with normal lymphocyte count.

*Conclusions*: FMF patients experience cardiac abnormalities during active inflammation, highlighting the importance of monitoring cardiac functions in these patients. Speckle tracking echocardiography can provide valuable insights into cardiac involvement in FMF. These findings emphasize the cardiac impact of FMF inflammation and the significance of long-term cardiac function monitoring in the management of FMF patients.

**Table:** 

**What is Known:** *• The current literature lacks studies investigating myocardial function in the pediatric population during the attack period of this particular disease.* *• Our objective was to assess the alterations in cardiac function during the attack and remission periods, considering clinical manifestations, disease severity, acute phase reactant levels, and mutation type. We also evaluated the pattern of cardiac involvement and the affected cardiac areas by comparing remission and attack periods*.	
**What is New:** *• Several studies have demonstrated a rise in the prevalence of ischemic cardiac disease and mortality among individuals with FMF.* *• Investigating cardiac involvement during the attack period in FMF patients can provide valuable insights for the prevention of long-term complications.*

**What is Known:**

*• The current literature lacks studies investigating myocardial function in the pediatric population during the attack period of this particular disease.*

*• Our objective was to assess the alterations in cardiac function during the attack and remission periods, considering clinical manifestations, disease severity, acute phase reactant levels, and mutation type. We also evaluated the pattern of cardiac involvement and the affected cardiac areas by comparing remission and attack periods*.

**What is New:**

*• Several studies have demonstrated a rise in the prevalence of ischemic cardiac disease and mortality among individuals with FMF.*

*• Investigating cardiac involvement during the attack period in FMF patients can provide valuable insights for the prevention of long-term complications.*

## Introduction

Familial Mediterranean fever (FMF) is an autosomal recessive, autoinflammatory disease with symptoms and clinical signs caused by serosal inflammation [1–3]. The disease is rare worldwide; however, in the Turkish population, its prevalence varies between 1/400 and 1/1000 [4, 5].

The most common forms of cardiac involvement in patients with FMF are pericarditis and pericardial effusion. There are a number of studies that have assessed the pericardium and endocardium in FMF patients during the attack period; however, there are few studies that have investigated myocardial function. It is thought that diastolic functions deteriorate in long-term disease follow-up [6, 7]. Interestingly, in adult patients with FMF in the remission period, there is no difference in systolic function compared to the healthy group, whereas diastolic dysfunction is present in both the right and left ventricles [7–11]. Finally, patients with FMF show cardiac degenerative changes including endothelial damage and an overall decline in cardiac function which result in cardiovascular morbidities.

In this study, we sought to characterize the changes in cardiac function and the impact of inflammatory acute phase reactants on these changes that occur during the active inflammation period and the remission period.

What is Known: . .

## Materials and methods

### Study population

In this study, we analyzed a cohort of 38 patients followed at the Istanbul University-Cerrahpasa Medical Faculty Hospital. This cohort consisted of patients diagnosed with FMF according to Turkish Pediatric FMF criteria. Patients without a *MEFV* gene analysis were not enrolled in the study. We excluded patients with a history of additional cardiac disease and/or amyloidosis and with less than a 1-year follow-up period.

FMF patients admitted to our hospital with an FMF attack were randomized to treatment with on-demand anakinra or treatment as usual (NSAIDs) based on their admission day and hours. Anakinra packages of the patients, whose therapy has been terminated or switched to another therapy because of ineffectiveness or adverse event, are stored in the refrigerator at the pediatric rheumatology outpatient clinic. There is a local protocol for the management of FMF attacks in our hospital. Our pediatric rheumatology outpatient clinic operates from Monday to Thursday from 08:00 am to 16:00 pm, thereby anakinra is available only for FMF patients who are admitted to the hospital with an attack during the daytime. Since there is no available anakinra at night shifts or at weekends, FMF patients admitted to the emergency clinic during these time frames were treated by NSAIDs. Demographic characteristics, clinical symptoms, family history, and *MEFV* gene analysis results were retrospectively obtained from patient files. The patients were examined, and blood tests, blood pressure measurements, and electrocardiogram evaluations were performed both during the attack and the remission periods. Additionally, conventional and speckle tracking echocardiographic evaluations were performed during the attack and remission periods. To differentiate if patients were in the attack or remission period, complete blood count, C-reactive protein level, and erythrocyte sedimentation rates were evaluated. In addition, troponin, B natriuretic peptide, and creatine kinase MB isoenzyme levels were re-measured during both periods. Patients with normal acute phase markers were not included in the cohort. During follow-up in remission periods, speckle tracking echocardiographic evaluation for the patients who had elevated acute phase markers was postponed until these markers were found to be within the normal range.

### Echocardiographic imaging

#### Conventional echocardiography

Transthoracic echocardiography was performed in the Pediatric Cardiology Department using a commercially available echocardiography machine (EPIQ CVx, Philips Medical Systems) equipped with an X5-1 MHz transducer. The echocardiographic examination was performed in the left lateral decubitus/supine position. A standard trans-thoracic echocardiogram was used in M-mode with two-dimensional Doppler flow assessments and tissue Doppler imaging. All pulsed-wave Doppler and tissue Doppler imaging parameters were measured at a sweep speed of 100 mm/s at the end of expiration, and the average of three consecutive heartbeats was recorded. All measurements were performed according to the recommendations of the American Society of Echocardiography. The left ventricular ejection and shortness fractions were calculated using the Teichholz formula [12]. Right ventricular functions were analyzed with right ventricular fractional area change. Right ventricular fractional area change represents a “surrogate” measurement of RV ejection fraction and is expressed as the percentage change in the right ventricular chamber area from end-diastole to end-systole. Tricuspid annular plane systolic excursion is a parameter of global right ventricular function that describes apex-to-base shortening [13]. The recommended methods for the assessment of ventricular diastolic dysfunctions are mitral valve and tricuspid valve inflow measurements from Doppler scan recordings. These recordings consist of peak early diastolic velocity (*e* wave), peak late diastolic velocity (*a* wave, atrial filling), and calculation of the *e*-to-*a* ratio for each ventricle. Tissue Doppler echocardiography was performed to measure peak early and late diastolic flow velocities (*e*′ and *a*′, respectively, cm/s) in the lateral tricuspid/mitral annulus views. In normal function, the *e* wave is bigger than the *a* wave, but with impaired relaxation, the *e*-to-*a* ratio will fall because of the increasing atrial filling wave (*a* wave).

#### Speckle tracking echocardiography (strain imaging)

**Fig. 1:**
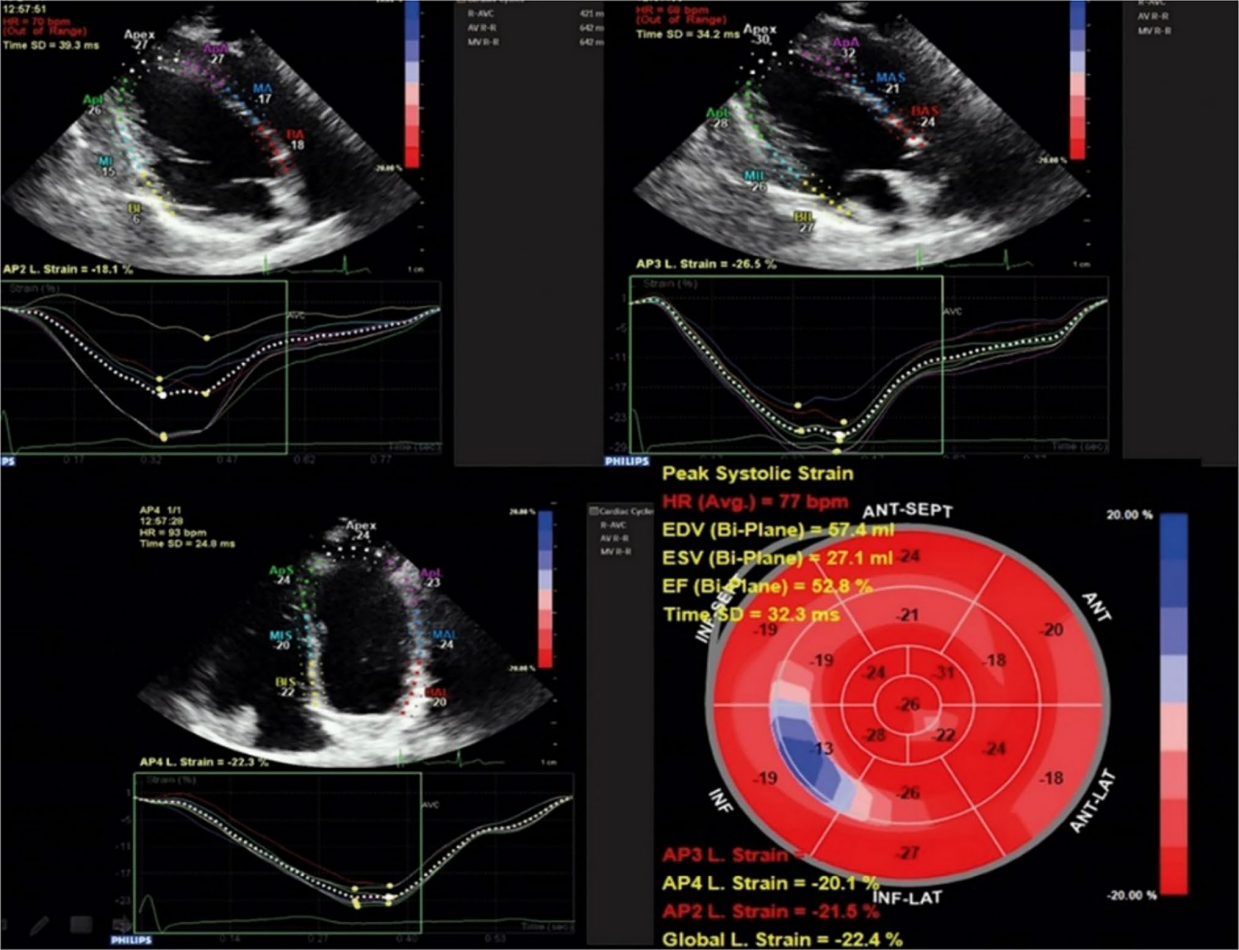
Speckle tracking strain echocardiography segmental analysis of cardiac function

Speckle tracking echocardiography is a new technique that analyzes motion by tracking natural acoustic reflections within an ultrasonic window. “Speckles” are stable patterns composed of 20–40 pixels that are automatically tracked during the cardiac cycle to follow the myocardial motion and directly assess the ventricular deformation at regions of interest [14]. Longitudinal left ventricular mechanics are the most sensitive components of the left ventricular dynamics, and these components are most sensitive to the presence of myocardial disease [15]. Therefore, we measured global longitudinal strain using speckle tracking echocardiography from three ultrasonic views. We acquired three consecutive beats using high frame rate harmonic imaging in each echocardiographic view. Cardiac cycles were recorded as two-dimensional color video loops, and the acquired raw data were saved for offline analysis (QLab; Philips Medical Systems) (Fig. 1). Manual adjustment of the regions of interest was performed as necessary. The average of each measurement was calculated.

### Assessment of disease severity

Pras scoring system was used to determine disease severity, and patients were divided into 3 subgroups according to their score [16, 17]. Patients with a score of 3–5 points were included in the mild disease activity group; 6–8 points were included in the moderate disease activity group; and 9 points or more were included in the severe disease activity group. Since there were only 2 patients with severe disease activity, these patients were excluded from the statistical analysis.

### Statistical analysis

The data were analyzed using the SPSS 23 statistical package program. The conformity of the data to normal distribution was confirmed by using the Kolmogorov–Smirnov test, and the homogeneity of variances was proved via Levene’s test. The mean difference comparisons of the measured parameters between the groups were tested by independent *t* test, and the mean difference comparisons of the intra-group control-attack measurements were tested by dependent *t* test. Chi-square test was used to analyze categorical data. Results are presented as mean ± standard deviation. Results were considered statistically significant for *p* < 0.05.

## Results

### Demographic characteristics

Eighteen female and 20 male patients were included in the cohort. The mean age of the patients in the cohort was 12.8 ± 4.8 years. The mean time to diagnosis was 15.2 ± 12.9 months in females and 22.8 ± 22.1 months in males, and no significant difference was found in the time to diagnosis (*p* = 0.204). The mean disease follow-up was 6.8 ± 4.7 years.

Of 38 patients, 13 (34%) had a consanguineous marriage between their parents. In 31 (81.6%) of the patients, there was a family history of a relative diagnosed with FMF.

Patients were divided into 7 different groups according to *MEFV* mutation type. Only two of our 38 patients had no *MEFV* mutation. Patients without a *MEFV* gene analysis were not enrolled in the study. The most frequent *MEFV* mutation was M694V homozygous, detected in 15 patients, followed by M694V heterozygous in 4 patients. M694V allele was detected in 19 (50%) of our patients.

### Laboratory findings and disease severity

The mean Pras score of the cohort was 6.1 ± 1.7. The mean Pras score was 5.7 ± 1.2 in males and 6.5 ± 2.1 in females. There was no significant difference in disease severity between genders. No significant difference between genders was noted (*p* = 0.144).

Patients with a score of 3–5 points were included in the mild disease activity group; 6–8 points were included in the moderate disease activity group; and 9 points or more were included in the severe disease activity group. In addition, no significant difference was found between genders in any of the groups according to this Pras score.

Of the laboratory tests obtained, leukocyte count, neutrophil count, C-reactive protein level, and erythrocyte sedimentation rate were found to be significantly higher in the attack period compared to the remission period (*p* < 0.005).

**Fig. 2:**
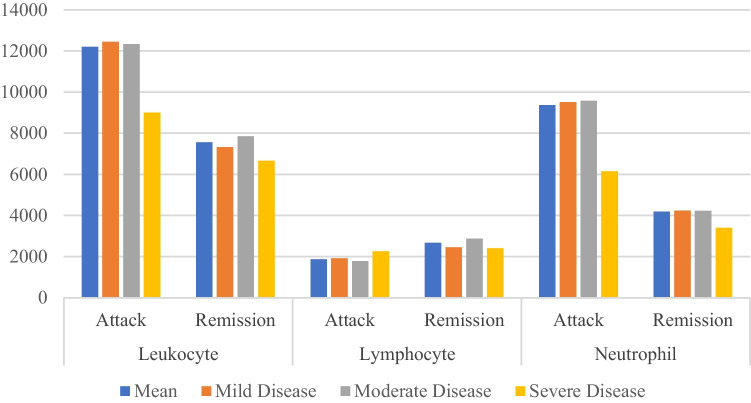
Leukocyte, lymphocyte, and neutrophil levels between the attack and remission periods

During the attack period, the mean leukocyte count, 12200.0 ± 4740.8/mm3; the neutrophil count, 9360.5 ± 4204.3/ mm3; and the ESR, 25.4 ± 12.5 mm/h were measured, while in the remission period, these values were 7550.0 ± 2040.1/mm3, 4189.5 ± 1951.8/mm3, and 11.2 ± 6.2 mm/h, respectively. In all parameters, the measurements in the attack period were significantly higher compared to the remission period (*p* = 0.0001). The laboratory findings of patients divided into three groups (mild, moderate, and severe) according to the Pras scoring system were compared, taking into account the severity of the disease. Comparative leukocyte and neutrophil levels were presented for the attack and remission periods in Fig. 2.

**Fig. 3:**
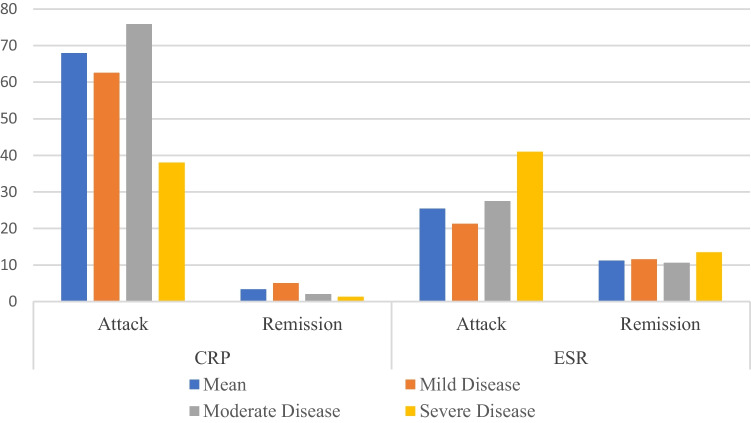
ESR and CRP levels between the attack and remission periods

CRP was measured at 98.9 ± 210.1 (median 48.0) in the attack period and at 14.6 ± 18.1 (median 7.5) in the remission period. A statistically significant difference was found between the attack and remission periods (*p* < 0.05). The changes in ESR and CRP levels, based on the disease groups, are shown in Fig. 3.

During the attack period, the lymphocyte count was measured to be 1863.2 ± 1040.4/mm3, whereas it was 2663.2 ± 895.5/mm3 during the remission period. A significant decrease in lymphocyte count was observed during the attack period compared to the remission period (*p* = 0.0001). In our study, a lower limit of 1500/mm3 was defined for lymphopenia. Among the 38 patients evaluated during an attack, 14 had lymphocyte counts below the normal range, and eight of them had lymphocyte values < 1000/mm3. In this group of patients with lymphopenia (14 patients), only two were receiving biologic agents regularly (anakinra, canakinumab). During the remission period, the lymphocyte counts of patients who experienced lymphopenia during the attack returned to the normal range (Fig. 2).

There was no significant difference between platelet count and ProBNP values measured in the attack and remission periods in relation to disease activation (*p* > 0.005).

No statistically significant difference was observed in the mean values of acute phase markers and blood count measurements between the mild and moderate disease groups (*p* > 0.05). The schematic representation shows the comparison of measurements among the disease groups and all patients collectively (Figs. 2 and 3).

According to Pras scoring, ECG findings of the mild and moderate disease groups in the attack and remission periods were compared. In the mild group, the heart rate was 102.2 ± 20.2/min, the PR interval was 136.5 ± 14.6/ms, and the QRS width was 83.5 ± 7.0/ms in the attack period, while it was 82.2 ± 12.5/min, 135.9 ± 18.4/ms, and 83.2 ± 8.5/ms in the remission period, respectively. There was a significant difference between the attack and remission periods in the mild disease group (*p* < 0.05).

In the moderate disease group, the heart rate was 111.7 ± 24.7/min, the PR interval was 134.7 ± 23.4/ms, and the QRS width was 80.5 ± 12.2/ms during the attack period, and 84.1 ± 17.7/min, 128.9 ± 18.2/ms, and 83.2 ± 9.5/ms during the remission period, respectively. There was a significant difference between attack and remission periods in the moderate disease group (*p* < 0.05).

When ECG changes of mild and moderate disease groups were compared with each other, no significant difference was found between the 2 groups (*p* > 0.05).

A significant difference was found in the mean durations of *e*-wave deceleration in the conventional echocardiographic evaluations between the attack (mean: 161.61 ± 46.0 ms) and remission (mean: 188.32 ± 41.1 ms) periods (*p* = 0.046). In conventional echocardiography measurements, no significant change was found between the attack and remission periods in parameters other than early deceleration time (*p* > 0.05).

During the attack period, speckle tracking echocardiographic evaluation demonstrated impaired function in the inferior segments of the left ventricle (Table 1).

**Table 1:** Evaluation of FMF patients in the attack and remission period with speckle tracking echocardiography

**Echocardiography parameters**	**Attack mean ± SD**	**Remission mean ± SD**	***p* value**
GLS_4Chs (%)	20.02 ± 5.6	22.33 ± 4.2	0.052
Basal inferoseptum	20.02 ± 5.9	23.34 ± 9.7	0.129
Midinferoseptum	21.92 ± 5.6	22.07 ± 5.1	0.910
Apicalseptum	17.51 ± 9.7	21.07 ± 7.9	0.108
Basalanterolateral	31.89 ± 11.4	33.52 ± 10.2	0.554
Midanterolateral	17.59 ± 6.6	19.03 ± 7.2	0.434
Apical lateral	13.37 ± 9.1	18.21 ± 8.1	**0.047**
GLS_2Chs (%)	17.04 ± 4.9	21.76 ± 4.5	**0.003**
Basalinferior	19.41 ± 8.4	24.66 ± 9.5	**0.003**
Midinferior	18.15 ± 7.6	20.55 ± 9.1	0.337
Apicalinferior	15.92 ± 6.9	20.71 ± 7.1	**0.023**
Basalanterior	19.14 ± 8.1	21.93 ± 7.2	0.236
Midanterior	16.74 ± 6.0	19.57 ± 7.1	0.184
Apicalanterior	16.54 ± 6.9	20.31 ± 7.4	0.100
GLS_3Chs (%)	18.11 ± 5.0	21.38 ± 3.0	**0.007**
Basalinferolateral	23.68 ± 8.7	32.97 ± 12.0	**0.001**
Midinferolateral	18.99 ± 6.9	19.1 ± 6.7	0.954
Apicallateral	13.35 ± 6.5	16.0 ± 6.2	0.097
Basalanteroseptum	23.33 ± 9.7	25.04 ± 9.1	0.495
Midanteroseptum	17.46 ± 7.6	18.97 ± 5.7	0.405
Apicalseptum	12.45 ± 6.3	16.19 ± 5.3	**0.017**

*GLS_2Chs* two-chamber view longitudinal strain, *GLS_3Chs* three-chamber view longitudinal strain, *GLS_4Chs* four-chamber view longitudinal strain

In Speckle echocardiographic evaluation, when mild and moderate disease groups were compared, the apical inferior segment was 13.8 ± 6.2 in the mild disease group and 18.3 ± 7.2 in the moderate disease group. There was a significant correlation with disease activity in the apical inferior segment (*p* < 0.05).

The mid-anterior segment measured 14.6 ± 5.7 in the mild disease group and 18.4 ± 6.1 in the moderate disease group. There was a significant difference between mild and moderate disease severity in the mid-anterior segment (*p* < 0.05). There was no significant difference in disease severity in other segments except these two segments (*p* > 0.05).

**Fig. 4:**
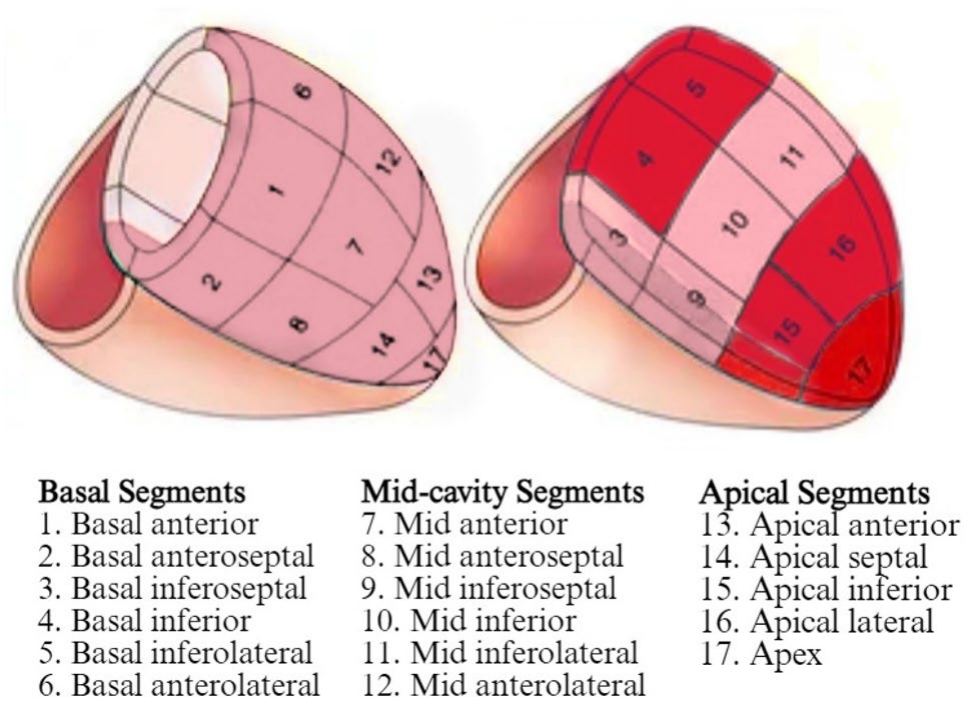
Affected cardiac segments during an attack

An assessment of these affected regions showed that right ventricular function was more affected in the moderate disease group. The affected regions are marked in red in Fig. 4.

In patients with lymphopenia, it was observed that the right ventricular function was more severely affected during the attack phase compared to patients with normal lymphocyte count. The change in TAPSE measurements between the attack and remission periods was − 16.1 ± 6.9% in the patients with lymphopenia during the attack and 3.7 ± 23.9% in the patients without lymphopenia. Statistically significant difference between the mean measurements was found (*p* = 0.019).

### Comparison of attack-remission changes according to attack treatment with speckle tracking echocardiography

Speckle tracking echocardiography evaluations were compared between the patients who received anakinra treatment during the attack (17 patients) and the patients who received only symptomatic treatment (21 patients). The percentage change between the attack and remission period in the measurement of the three-chamber basalinferolateral segment in the patients who received only symptomatic treatment during the attack was 84.5 ± 96.1%. Meanwhile, the change between the attack and remission period in the patients who received anakinra was 20.8 ± 51.3%. A significant difference was observed between the two groups (*p* = 0.041).

## Discussion

In this study, we sought to establish a relationship between known markers of cardiac function and standard laboratory tests that could be easily accessed. Given our institutional access, we had a unique opportunity to obtain patient data and clinical assessment tools not easily found outside of our center. As such, this singularly positioned us to be able to ask more probing questions regarding the relationship between physiologic and anatomic changes that we see in FMF patients. We determined that this would be important, as it would help provide a clinically relevant tool that can then be re-applied to patient care without necessitating additional changes in protocol. As such, we demonstrated that there were shifts in cardiac function as noted prior and showed that this correlated to shifts in neutrophil counts, C-reactive protein, and erythrocyte sedimentation rate.

Of particular note, there is a marked decrease in left and right ventricular function in FMF patients. This is especially apparent in the attack period in the left inferior segments. In addition, we found that right ventricular systolic function decreased more in the moderate disease subgroup in comparison to the mild disease subgroup (Table 1). Interestingly, there is a concurrent decrease in lymphocytes in the attack period compared to the remission period. In a previous study, 53% of the relatives of patients with FMF had a history of relatives diagnosed with FMF [1]. In our study, unlike the literature, 31 of our 38 patients (81.6%) had a family history of a relative diagnosed with FMF.

In autoinflammatory diseases, acute-phase reactants were found to be significantly higher during the attack period compared to the remission period. In some patients, acute-phase reactants were reported to be elevated in the absence of clinical findings due to the persistence of subclinical inflammation [1, 4, 5]. Similar to the findings in the literature, C-reactive protein and erythrocyte sedimentation rate measurements were found to be significantly higher in the attack period compared to the remission period in our patient group when the attack and remission periods were considered. Again, similar to the findings in the literature, leukocyte and neutrophil values measured in our patients were significantly higher in the attack period compared to the control period.

According to prior studies, neutrophil production increases in response to cytokines and proteins formed during attack in inflammatory diseases. Due to this increase, the neutrophil/lymphocyte ratio also increases. Stress is known to be a triggering factor for attacks in patients with FMF due to its increasing effect in the cortisol levels which leads to a decrease in lymphocyte count (3). In our comparative analysis of blood counts, a significant decrease in lymphocyte count in the attack period was observed. It showed that the lymphocyte count reverted to the normal range during the remission period. Since there are no prior studies in terms of the subject, no external data comparison has been performed. In this case, as a limiting point, we could not biochemically evaluate stress hormones, cortisol levels, and cytokine levels in our patients during the attack period. We think that this situation can be clarified with similar studies to be conducted in the future in patients with FMF.

We used speckle tracking echocardiography instead of conventional echocardiography. The primary advantage of this method is its reduced dependence on both the load and the Doppler angle of incidence compared to conventional methods. This technique involves measuring the displacement of speckles on a 2-dimensional echocardiographic image. Speckle tracking provides directional independence of the ultrasound beam and illustrates myocardial deformation within the segment of interest, rather than the volumetric changes observed in conventional echocardiography [18, 19]. In the speckle tracking echocardiographic evaluation of the patients, statistically significant differences were found in the global longitudinal two-chamber view, inferior and anterior sections when the attack and remission periods were compared. This suggests that these differences are related to the cytokines that are released during the inflammation period. Studies on how increased TNF-α and IL-1β levels during an attack may affect cardiac functions have shown that increased TNF-α expression decreases endothelium-dependent vasodilation in coronary arterioles. The mechanism responsible for this is decreased nitric oxide bioavailability and nitric oxide-dependent vasodilation [20, 21]. Experimental studies have shown that injection of recombinant IL-1β (3 µg/kg) in experimental mice causes a decrease in myocardial contractility and systolic dysfunction [22, 23].

In clinical studies, a single dose of 100 mg anakinra given to patients with rheumatoid arthritis increased coronary flow reserve by affecting myocardial contractility and relaxation through IL-1 blockade within 3 h [24]. In our evaluation of the speckle tracking echocardiographic measurements, we observed that patients who received anakinra treatment during the attack demonstrated faster improvement of the heart function in the three-space basal inferolateral region compared to patients who did not receive anakinra.

In our study, we were not able to determine whether the decrease in left ventricular function during an attack period was due to the effect of inflammatory cytokines on the microvascular environment, or the changes in coronary flow velocity. In the healthy population, because of the variation in the coronary arteries that perfuse the posterior and inferior regions of the left ventricle, coronary flow velocity could not be associated with the involvement of these regions during an attack [25].

Conventional echocardiographic measurements (TAPSE) during the attack period for patients with normal lymphocyte counts and lymphopenia showed a significant difference in right ventricular function. Moreover, TAPSE evaluations have shown that the patients with lymphopenia demonstrated more changes than the patients with normal lymphocyte count when attack and remission periods were compared. Furthermore, the speckle tracking echocardiographic evaluation showed a significant alteration in the three-space midinferolateral area for the patients with lymphopenia.

We think that right ventricular systolic function was more affected in the lymphopenia group due to increased cytokines and inflammation. We believe that future studies in different autoinflammatory diseases will clarify this.

## Conclusion

We demonstrated that cardiac function decreased in the left ventricular inferior segments during the attack period in our study group. We found that the systolic function of the right ventricle decreased more in the moderate disease group than in the mild disease group during the attack period. We also found that the patient group with lymphopenia during the attack had decreased right ventricular function compared to the normal group.

## Limitations

1. We evaluated our patient group within the 24–72-h period of cardiac and biochemical findings during the attack period of the disease. We think that cardiac functions may have been detected differently in some patients because our patients were evaluated within 48 h. We think that the results would be more meaningful if we could evaluate our patient group in a shorter and more specific period during the attack process.

2. We assumed that serum amyloid A, TNF alpha, and IL 1 measurements may be more effective in evaluating the degree of inflammation during the attack period when we evaluate the cardiac involvement of our patients. In light of this study, we plan to take these limitations into consideration in future studies.

## Data Availability

The data that support the findings of this study are available from the corresponding author upon reasonable request.

## Abbreviations

**CK-MB**: Creatine kinase-myocardial band
**CRP**: C-reactive protein
**ESR**: Erythrocyte sedimentation rate
**FMF**: Familial Mediterranean fever
**GLS_2Chs**: Two-chamber global longitudinal strain
**GLS_3Chs**: Three-chamber global longitudinal strain
**GLS_4Chs**: Four-chamber global longitudinal strain
**IL**: Interleukin
**MEFV**: Mediterranean fever
**Pro-BNP**: Pro–B-type natriuretic peptide
**TAPSE**: Tricuspid annular plane systolic movements
**TNF**: Tumor necrosis factor
